# Short-term fasting enhances the resistance of common carp (*Cyprinus carpio*) to *Aeromonas hydrophila*: Impacts on gut microbiota, glucose, and oxidative stress

**DOI:** 10.14202/vetworld.2025.1955-1963

**Published:** 2025-07-17

**Authors:** Dahliatul Qosimah, Tiara Widyaputri, Muhamad Firman Ataullah, Syifa Fu’ada, Zackya Najib, Atsir Farhan, Fathia Zahra Aulia Putri, Anggieta Ratuyustiarany

**Affiliations:** 1 Laboratory of Veterinary Microbiology and Immunology, Faculty of Veterinary Medicine, Universitas Brawijaya, Malang, East Java 65151, Indonesia; 2 Veterinary Clinical Pathology and Experimental Animal Laboratory, Faculty of Veterinary Medicine, Universitas Brawijaya, Malang, East Java 65151, Indonesia; 3 Faculty of Veterinary Medicine, Universitas Brawijaya, Malang, East Java 65151, Indonesia

**Keywords:** *Aeromonas hydrophila*, common carp, fasting, glucose, hemoglobin, lactic acid bacteria, oxidative stress

## Abstract

**Background and Aim:**

*Aeromonas hydrophila* is a significant pathogen in freshwater aquaculture, contributing to high morbidity and mortality in common carp (*Cyprinus carpio*). Conventional reliance on antibiotics raises concerns about resistance and environmental impact. This study aimed to evaluate the effects of short-term fasting (1 or 2 days) on physiological, oxidative stress, and microbial responses in *C. carpio* infected with *A. hydrophila*.

**Materials and Methods:**

Sixty *C. carpio* were divided into four groups (n = 15): negative control (uninfected), positive control (infected), T_1_ (1-day fasting + infected), and T_2_ (2-day fasting + infected). Infections were induced by immersion in an *A. hydrophila* suspension (10^8^ colony-forming units [CFU]/mL). Three days post-infection, blood was collected for glucose and hemoglobin analysis. Malondialdehyde (MDA) levels in head kidney tissue were assessed as a marker of oxidative stress. Gut samples were analyzed for lactic acid bacteria (LAB) through standard plate counts. Statistical comparisons were made using a one-way analysis of variance and Kruskal–Wallis tests (p < 0.05).

**Results:**

The T_2_ group (2-day fasting) exhibited significantly better physiological responses than T_1_ and the positive control. Blood glucose levels in T_2_ (83.5 ± 1.71 mg/dL) were significantly lower than the positive control (127 ± 3.85 mg/dL), but within the normal range. Hemoglobin levels were highest in T_2_ (7.8 ± 0.27 g/dL), indicating preserved oxygen-carrying capacity. MDA levels, though not statistically different, were lowest in T_2_ (14.42 ± 0.60 mg/L), suggesting reduced oxidative stress. LAB counts were highest in T_2_ (1.69 × 10^9^ CFU/g), indicating improved gut microbiota balance.

**Conclusion:**

A 2-day fasting regimen enhanced disease resistance in *C. carpio* by modulating glucose metabolism, preserving hematological integrity, reducing oxidative stress, and enriching beneficial gut microbiota. These findings support short-term fasting as a promising non-pharmacological strategy for managing bacterial infections in aquaculture, with the potential to reduce antibiotic dependence.

## INTRODUCTION

The common carp (*Cyprinus carpio*) is a significant freshwater aquaculture species in Indonesia, contributing substantially to the country’s overall fish production [1]. However, the intensification of aquaculture systems has increased the prevalence of infectious diseases, particularly those caused by opportunistic pathogens such as *Aeromonas hydrophila*. This bacterium is the primary causative agent of motile *Aeromonas* septicemia, characterized by hemorrhaging, systemic inflammation, and high mortality in fish populations [2]. Although antibiotics are commonly used to manage *A. hydrophila* infections, their overuse poses serious concerns, including antimicrobial resistance, environmental disruption, and the presence of drug residues in fish intended for human consumption [3, 4].

To address these challenges sustainably, non-antibiotic approaches have gained interest due to their proven effectiveness and low ecological footprint. One such method involves the strategic use of short-term fasting to modulate the fish’s nutritional status. Fasting is known to affect various physiological parameters in fish, including metabolic activity, stress responses, immune function, gut microbial composition [5, 6], and antioxidant mechanisms [7]. Karatas *et al*. [8] reported that short-term fasting improves energy efficiency, reduces free radical production, and helps reestablish oxidative balance. Additionally, fasting can affect the quantity and diversity of beneficial gut microbes, particularly lactic acid bacteria (LAB), which play a crucial role in mucosal defense and systemic immune modulation [9, 10].

Previous studies by Qosimah *et al*. [11] and Effendi *et al*. [12] have demonstrated that bacterial infections, such as *A. hydrophila*, increase blood glucose levels through stress-induced gluconeogenic activity [13]. This hyperglycemic state can disrupt immune balance and trigger oxidative stress [14]. Malondialdehyde (MDA), a lipid peroxidation product, is widely recognized as an indicator of oxidative damage resulting from infection or environmental stressors [15]. Hemoglobin serves as a marker of blood health and oxygen transport capacity, which may decline due to infection-related inflammation and erythrocyte degradation [16, 17]. While oxidative stress induced by *A. hydrophila* infection can cause significant tissue and cellular damage, the role of short-term fasting as a non-pharmacological approach to modulate immune-metabolic responses in *C. carpio* remains insufficiently investigated.

Although *A. hydrophila* infections pose a significant threat to freshwater aquaculture species, such as *C. carpio*, sustainable and non-pharmacological disease management strategies remain underexplored. Short-term fasting has shown promise in modulating stress responses, metabolism, immune function, and gut microbiota in various fish species. However, empirical evidence remains limited regarding its effectiveness in enhancing resistance to *A. hydrophila*, specifically in common carp, particularly in relation to hematological parameters, oxidative stress markers, and gut microbial dynamics. This leaves a critical gap in understanding how fasting-induced physiological adaptations could be leveraged to improve host resilience during bacterial infections.

This study aimed to investigate the effects of short-term fasting on the physiological and microbial responses of *C. carpio* infected with *A. hydrophila*. Specifically, it assessed how 1-day and 2-day fasting regimens influence blood glucose levels, hemoglobin concentration, oxidative stress (as measured by MDA levels), and the abundance of LAB in the gut. By evaluating these parameters, the study seeks to determine whether controlled fasting could serve as a viable non-antibiotic intervention to enhance disease resistance and promote immune-metabolic balance in common carp under infectious stress.

## MATERIALS AND METHODS

### Ethical approval

Ethical clearance for this study was granted by the Universitas Brawijaya Ethics Committee under approval number 173-KEP-UB-2024.

### Study period and location

The study was conducted from October to December 2024 at the Faculty of Veterinary Medicine, Universitas Brawijaya, Malang, Indonesia

### Study design and experimental setup

This laboratory-based study employed a post-test only control group design, with sample size determined using a completely randomized design.

Sample handling and analyses were conducted in the following laboratories: The Veterinary Microbiology and Immunology Laboratory (bacteriological testing), the Clinical Pathology Laboratory (hematological testing), and the Fish Disease and Parasitology Laboratory (fish maintenance), all located within the Faculty of Fisheries and Marine Sciences at Universitas Brawijaya.

### Experimental animals and grouping

Sixty *C. carpio* (11–13 cm in length, ~35 g) were acclimated for 7 days in tanks with continuously aerated, clean water maintained at 26°C–28°C and pH 7.0–7.5 [18]. After acclimatization, the fish were randomly allocated into four treatment groups (n = 15/group; three replicates of five fish each):

- Negative control: No fasting, no infection
- Positive control: Infected only
- T_1_: 1-day fasting followed by infection
- T_2_: 2-day fasting followed by infection

### Fish husbandry

Each 30-L tank contained 25 L of dechlorinated water and housed five fish [19]. Tanks were disinfected with 50 ppm chlorine for 24 h, followed by neutralization with 350 ppm sodium thiosulfate for another 24 h [20]. Water was continuously aerated [21], and the fish were fed commercial pellets (PT Japfa Comfeed, Indonesia) twice daily during the acclimation period. A 10-day acclimatization period preceded the treatments.

### Fasting and infection procedure

Fish in the positive control, T_1_, and T_2_ groups were fasted for 0, 1, or 2 days, respectively, then infected by immersion in *A. hydrophila* suspension (10^8^ colony-forming units [CFU]/mL) under aeration. The negative control group was immersed in pathogen-free well water. All fish were observed for 3 days post-infection.

### Bacterial culture preparation

*A. hydrophila* was cultured on Rimler-Shotts medium (Merck, Germany), a selective agar containing yeast extract (3.0 g/L), maltose (3.5 g/L), L-cysteine hydrochloride (0.3 g/L), L-lysine hydrochloride (5.0 g/L), L-ornithine hydrochloride (6.5 g/L), sodium thiosulfate (6.8 g/L), ferric ammonium citrate (0.8 g/L), sodium deoxycholate (1.0 g/L), sodium chloride (5.0 g/L), bromothymol blue (0.03 g/L), and agar (13.5 g/L). The plates were incubated for 24 h at 27°C.

### Clinical observation and sampling

Following infection, fish were monitored for signs of illness, including hemorrhaging, erratic behavior, fin erosion, and mortality [22]. After 72 h, blood samples were collected from the caudal vein using a 1 mL syringe with a 26-G needle without anesthesia [23].

### Blood biochemical analysis

Plasma glucose was measured using a Gluco Dr. Auto meter with gold-plated strips (All Medicus Co., Ltd., Germany) [24]. Hemoglobin concentration was determined using a Sahli-type hemometer (Marienfeld, Germany). For necropsy, fish were anesthetized by immersion in a solution of 200 mg/L MS-222 and 400 mg/L NaHCO_3_ [25].

### Gut microbiota enumeration

Intestinal segments were aseptically removed and homogenized (10% w/w) in 0.85% saline. Samples were incubated in de Man, Rogosa, and Sharpe (MRS) broth (Merck KGaA, Germany) [26], followed by serial dilution (10^−1^–10^−9^) and plating on MRS agar. Plates were incubated at 37°C for 48 h under microaerophilic conditions using a GasPak system (BD, USA). LAB colonies were counted digitally and reported as CFU/g. Valid colony counts ranged from 25 to 250 [27].

### Oxidative stress assessment

Head kidney tissue (~100 mg) was homogenized in 900 μL of 1.15% potassium chloride for the determination of MDA using the thiobarbituric acid (TBA) reactive substances method. A 25 μL aliquot was mixed with 200 μL of 20% acetic acid (pH 3.5), 200 μL of 8.1% sodium dodecyl sulfate, and 200 μL of 0.8% TBA, then incubated at 95°C for 60 min. After cooling, 1.5 mL of a 15:1 (v/v) n-butanol: Pyridine mixture was added. Samples were centrifuged at 800 × *g* for 10 min, and the organic phase was collected. Absorbance was read at 532 nm using a UV-1800 spectrophotometer (Shimadzu, Japan), and MDA concentrations were calculated from a 1,1,3,3-tetramethoxypropane standard curve [28].

### Statistical analysis

Data normality was assessed using the Shapiro–Wilk test. Group comparisons were made using a one-way analysis of variance, followed by Tukey’s *post hoc* test for pairwise differences. Statistical significance was set at p < 0.05. Results are presented as mean ± standard deviation. Analyses were performed using GraphPad Prism version 10 (GraphPad Software, San Diego, CA, USA).

## RESULTS AND DISCUSSION

### Clinical symptoms and mortality

Exposure to *A. hydrophila* induces typical clinical signs of systemic infection in *C. carpio*, including labored breathing, hemorrhagic lesions on the skin, superficial wounds, and balance disturbances during swimming. These clinical manifestations are consistent with those reported by Patil *et al*. [29], who observed that rainbow trout (*Oncorhynchus mykiss*) exhibit lethargy, slow movements, and gasping before death due to infection. In the present study, symptoms were severe in the positive control group, mild in the T_1_ and T_2_ groups, and absent in the negative control group. These findings suggest that short-term fasting before infection mitigates the severity of disease symptoms. This trend aligns with Domínguez-Andrés *et al*. [30], who suggested that short-term fasting primes the immune system and enhances resilience to bacterial infection. Notably, fish in the T2 group exhibited better clinical outcomes than those in T1, supporting the idea that a longer fasting duration enhances adaptive preparedness. This observation also aligns with the study by Wang *et al*. [31], which demonstrated that short-term fasting (3 days) before infection can enhance the resistance of non-vaccinated Nile tilapia to *Streptococcus agalactiae*. However, this contrasts with Caruso *et al*. [32], who found that fasting did not significantly affect the non-specific immune response in red porgy fish. Conversely, resumption of feeding within a short period (7 days) was associated with elevated hemagglutination titers. The health of fish during infection heavily relies on the innate immune system, especially under suboptimal thermal conditions, as the proliferation of T and B cells and antibody production are impaired [33], depending on factors such as fasting duration, species, and age.

### Glucose metabolism and stress modulation

**Figure 1:**
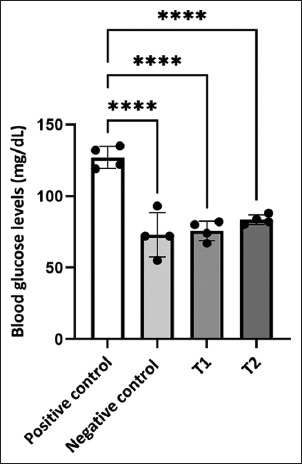
The effect of fasting and *Aeromonas hydrophila* infection on blood glucose levels. The results show that fasting can lower blood glucose levels caused by bacterial infection compared to the non-fasting control group, p < 0.0001.

Blood glucose concentrations (mg/dL) differed significantly across all treatment groups (p < 0.0001). The mean glucose levels in T_1_ (75.75 ± 3.38) and T_2_ (83.5 ± 1.71) remained within the physiological range. The positive control group exhibited the highest glucose concentration (127 mg/dL), which was significantly above the normal physiological range for common carp (40–90 mg/dL) [34], indicating a strong metabolic stress response to *A. hydrophila* infection (Figure 1). These results indicate that short-term fasting prom-otes efficient glucose utilization and adaptive stress responses.

The reduction in glucose levels reflects suppressed gluconeogenesis and glycogenolysis under nutrient-limited conditions. Modulation of host metabolism may represent a viable strategy for enhancing resistance to bacterial infections. The hyperglycemia observed in the infected and non-fasted group was consistent with stress-induced activation of gluconeogenesis and suppression of insulin-like signaling pathways. Tang *et al*. [35] demonstrated that stressors, such as low temperatures, can elevate blood glucose levels by enhancing phosphoenolpyruvate carboxykinase expr-ession and reducing pyruvate kinase activity, resulting in oxidative damage and impaired immune capacity. This suggests that excessive blood glucose is not only a stress biomarker but also a physiological disruptor that contributes to redox imbalance and immune dysfunction. In contrast, fasting groups (T_1_ and T_2_) showed improved glucose regulation. Although both groups maintained normoglycemia, T_1_ had slightly lower values than T_2_. However, considering the hematological and oxidative stress profiles, T_2_ demonstrated more comprehensive physiological stability. This supports the hypothesis that fasting duration influences adaptive capacity, with 2-day fasting providing a more balanced metabolic adjustment. Several studies have supported these findings. Mishra *et al*. [36] reported that Nile tilapia fasted for 3 days exhibited significantly reduced glucose levels. Nonetheless, blood glucose levels normalized following the refeeding period. Peng *et al*. [37] observed in *Megalobrama amblycephala* that a 2–3 day fast enhanced glycolysis and shifted amino acid catabolism toward energy maintenance, thereby improving resistance to transport-induced ammonia stress. On a molecular level, Ntantali *et al*. [38] found that fasting in sea bass modulated glycolytic gene expression (*hk, ldha*, and *gpi*), demonstrating that the metabolic response extends beyond blood parameters. In crustaceans, Tian *et al*. [39] further demonstrated that alternate-day fasting increased UDP-GlcNAc levels, a marker of nucleotide sugar metabolism, suggesting adaptive hyperglycemia in response to prolonged metabolic pressure. These molecular adaptations highlight the nuanced nature of fasting responses–transient glucose suppression may benefit fish in the context of acute infection, but excessive or prolonged fasting can lead to metabolic destabilization. Extended fasting studies reinforce this caution. Fernández-Muela *et al*. [40] reported that 20-day fasting in rainbow trout activated hepatic gluconeogenesis while reducing glycolysis, signaling energetic strain. Karatas *et al*. [8] demonstrated that prolonged fasting (70–120 days) reduced glucose levels but also depleted energy reserves, resulting in muscle atrophy and oxidative DNA damage. Collectively, these findings indicate that short-term fasting, particularly for 2 days, provides a window of metabolic optimization. It limits hyperglycemic stress while preserving energy balance, thereby enhancing the fish’s resilience against bacterial challenge with-out inducing the adverse effects of prolonged nutrient deprivation.

### Effects of fasting and infection on hemoglobin levels and hematological responses

**Figure 2:**
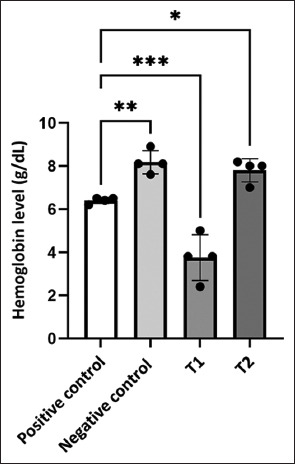
Hemoglobin level (g/dL) across treatment groups. Values are presented as mean ± standard deviation. The T_2_ group (2-day fasting) maintained significantly higher hemoglobin levels than the T_1_ and positive controls. Statistical differences are indicated by asterisks (*p < 0.05; **p < 0.01; ***p < 0.001).

Hemoglobin levels varied significantly among the groups (p < 0.0001), as shown in Figure 2. Hemoglobin levels were highest in the negative control group (8.18 ± 0.27 g/dL), followed by T_2_ (7.8 ± 0.27 g/dL), both within the normal physiological range for *C. carpio* (7.84–8.56 g/dL) [41]. These values indicate stable hematological function and strong oxygen-carrying capacity. In contrast, the significant decrease in hemoglobin levels in T_1_ (3.75 ± 0.53 g/dL) suggests impaired hematopoiesis or increased hemolysis due to the combined stress of infection and inadequate adaptation to fasting. The positive controls also showed moderately reduced hemoglobin (6.4 ± 0.7 g/dL), likely due to infection-related damage to erythrocytes.

The notably low T_1_ hemoglobin level may suggest incomplete physiological adaptation during fasting. *A. hydrophila* infection damages red blood cells, induces hemolytic anemia, and diminishes antioxidant defense mechanisms in fish [41]. Similar results were observed in Nile tilapia (*Oreochromis niloticus*) infected with *Shewanella putrefaciens*, where hemolysis and metabolic stress led to impaired hematopoiesis and changes in immune gene expression [42]. Interestingly, while short-term fasting has been associated with hematological improvements in some species, such as *Lophiosilurus alexandri* juveniles, the findings here indicate that a single day of fasting (T_1_) is not enough to achieve such benefits in *C. carpio*. This highlights that hematological responses to fasting vary between species and depend on fasting duration and immune status. In contrast, the T_2_ group had hemoglobin levels similar to those of the negative control, suggesting that two days of fasting provided enough time for physiological adaptation to help preserve erythrocyte integrity during infection. These findings emphasize that properly timed short-term fasting supports hematological resilience by maintaining red blood cell stability and oxygen delivery during pathogen exposure.

### Lipid peroxidation and oxidative stress

**Figure 3:**
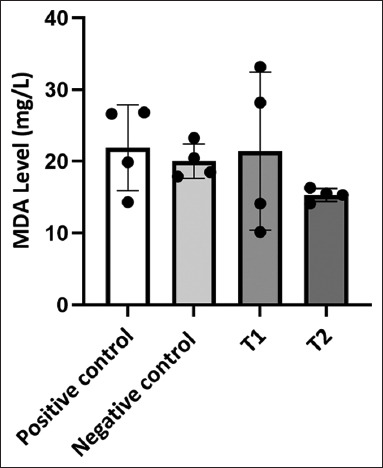
Malondialdehyde (MDA) levels (mg/L) in *Cyprinus carpio* between treatment groups. Although the differences were not statistically significant (p > 0.05), the T_2_ group exhibited the lowest MDA levels and reduced variability, indicating better oxidative stability compared with the other groups.

No statistically significant differences in MDA concentrations (mg/L) were observed between the groups (p > 0.05), although meaningful physiological trends were apparent (Figure 3). The highest ave-rage MDA was found in the positive control group (21.90 ± 3.00), followed by T_1_ (21.41 ± 5.50), the negative control (20.02 ± 1.21), and the lowest in T_2_ (14.42 ± 0.60). The elevated MDA levels in the positive control and T_1_ groups indicate that bacterial infection and inadequate metabolic adaptation contribute to increased oxidative stress. Conversely, the T_2_ group, which experienced 2 days of fasting, showed significantly lower MDA levels and less individual variation, suggesting a more stable oxidative environment and potentially enhanced antioxidant defense mechanisms.

MDA, a marker of oxidative stress, was highest in the positive control and T_1_ groups (1-day fasting), indicating that *A. hydrophila* infection and insufficient compensation of metabolic stress can cause significant oxidative damage. A single day of fasting was not enough to fully activate the body’s antioxidant defense system. This aligns with the idea that oxidative stress in fish infected with opportunistic bacteria results from the buildup of reactive oxygen species (ROS), mainly hydrogen peroxide (H_2_O_2_) and superoxide (O_2_^-^), which harm lipids, proteins, and nucleic acids through oxi-dative chain reactions [43, 44].

The lowest MDA levels were observed in the T_2_ group (2-day fasting), suggesting that a longer fasting period could offer protective effects against ROS buildup. This effect is probably due to increased activation of antioxidant defenses, including higher activity of antioxidant enzymes, as reported by Cheng *et al*. [45], who showed that fasting can suppress the mammalian target of rapamycin (mTOR) pathway, a key controller of autophagy and cell metabolism. Reduced mTOR activity triggers cellular defense mechanisms by boosting the expression of antioxidant enzymes, which then lowers oxidative levels, including MDA, in bacterial cells during infection. These findings differ from those by Bu *et al*. [46], who found that the activities of superoxide dismutase, catalase, and glutathione peaked on day 3 of fasting. Their results indicated that both enzymatic and non-enzymatic antioxidant systems are activated to fight ROS. Although statistical analysis did not show significant differences, the biological importance of lowered MDA in T_2_ supports the idea that 2-day fasting provides oxidative protection in infected *C. carpio*. This emphasizes the need to optimize fasting duration to balance immune response and oxidative control during bacterial infection.

### Gut microbiota modulation

Short-term fasting influenced the abundance of LAB in the intestinal tract of *C. carpio*. The highest LAB abundance was seen in the T_1_ group (1.53 × 10^9^ CFU/g), followed by T_2_ (1.29 × 10^9^ CFU/g). In comparison, the positive control (infected without fasting) had a lower LAB population of 1.05 × 10^9^ CFU/g, while the negative control showed the lowest count at 2.0 × 10^8^CFU/g. The elevated LAB counts in the T_1_ and T_2_ groups indicate that short-term fasting can influence gut microbiota, especially by boosting beneficial bacteria like *Lactobacillus* spp., which are vital for maintaining mucosal integrity, preventing pathogen growth, and producing antimicrobial metabolites and immune modulators [47].

These results align with Mohr *et al*. [9], who reported that intermittent fasting boosts the diversity and composition of the gut microbiota, including *Lacto-bacillus* spp., which support metabolic and immune health. Additionally, Messina *et al*. [48] showed that fasting can cause temporary dysbiosis; however, refeeding can restore a healthy microbiota, including Firmicutes and Bacteroidetes, thereby enhancing gut function and the balance of protective bacteria, such as LAB. Moreover, Li *et al*. [49] found that fermented Moringa oleifera leaves significantly improved gut microbiota balance, increasing the proportion of protective bacteria like Firmicutes and decreasing the population of pathogens, including Aeromonas, in Procambarus clarkii.

The LAB counts in the positive control group were lower than in all treatment groups, likely because there was no nutritional intervention or fasting, which can actively influence gut microbiota. Infection with *A. hydrophila*, without additional treatments, can disturb microbiota balance, including decreasing commensal bacteria like LAB, due to pathogen dominance and localized inflammation in the gastrointestinal tract [50]. Additionally, Mohr *et al*. [9] stated that without fasting cycles or caloric restriction, gut flora tends to stay more inflammatory and less protective, especially during immune challenge. Therefore, the differences in LAB populations between the positive control and fasting groups highlight the modulatory effect of fasting on gut microbial ecology, particularly through better fermentative conditions, pH control, and stimulation of the gut mucosa.

The 2-day fasting period (T_2_) seems to offer a more balanced protective effect by influencing microbiota, glucose metabolism, and oxidative response. The rise in LAB, along with stable biological parameters, supports the idea that fasting, when done for the right duration, can be an eco-friendly and sustainable way to manage fish health, providing a non-antibiotic alternative to fight bacterial infections like *A. hydrophila*.

Overall, previous studies support the idea that short-term fasting and natural substances are promising strategies for boosting fish resistance to bacterial infections and environmental stress. However, the complex interactions between fasting, microbiota, oxidative stress, and hematological responses seen in this study highlight the need for a more comprehensive approach in future research. In general, fasting has shown to be a promising non-drug strategy for increasing fish resistance to bacterial infections; however, the duration and the fish’s baseline physiological condition must be carefully considered.

Several limitations of this study should be taken into account when interpreting the results and planning future research. These include the short observation period. The study only examined effects for 2 days after infection, which may not be enough to fully understand the long-term immune and metabolic responses to fasting and bacterial infection. Additionally, immune parameters were limited; for example, humoral immune markers such as specific antibodies or cytokine expression were not measured, so immune mechanisms were not comprehensively explored.

## CONCLUSION

This study showed that a 2-day short-term fasting plan significantly improves the resistance of *C. carpio* to *A. hydrophila* infection. Important physiological changes were seen in the T_2_ group, including lower blood glucose levels (83.5 ± 1.71 mg/dL), higher hemoglobin concentrations (7.8 ± 0.27 g/dL), reduced MDA levels (14.42 ± 0.60 mg/L), and increased gut LAB levels (1.29 × 10^9^ CFU/g). These results indicate that short-term fasting helps regulate glucose, maintain blood health, reduce oxidative stress, and support a healthy gut microbiota – all of which help improve resistance to bacterial infections.

From a practical perspective, adding short-term fasting to aquaculture management strategies provides a non-drug, cost-efficient, and environmentally friendly way to boost fish health. This approach may also reduce reliance on antibiotics, helping to address issues related to antimicrobial resistance and drug residues in aquatic environments.

The main strength of this study is its comprehensive evaluation of metabolic, hematological, oxidative, and microbial parameters under controlled conditions, offering a detailed understanding of host-pathogen interactions affected by nutritional interventions. However, some limitations must be recognized. The observation period was only 2 days after infection, which might not reveal longer-term immunometabolic adaptations. Additionally, the study did not examine molecular or humoral immune markers (such as cytokine profiles and antibody titers), leaving gaps in under-standing the complete immune response to fasting.

Future studies should expand the observation period and incorporate molecular and transcriptomic analyses to clarify the underlying pathways connec-ting fasting to immune modulation. Exploring the synergistic effects of fasting with probiotics or phytogenic immunostimulants may further improve the effectiveness of these interventions. Additionally, field-scale validation is necessary to evaluate feasibility and outcomes in commercial aquaculture environments.

This study highlights the potential of controlled short-term fasting, specifically a 2-day regimen, as a strong and sustainable way to improve the health and disease resistance of common carp. This method meets the rising demand for eco-friendly aquaculture techniques and provides a promising path for enhancing fish welfare and production efficiency.

## AUTHORS’ CONTRIBUTIONS

DQ and TW: Conceptualized and designed the study. DQ, TW, AF, AR, FZAP, MFA, SF, and ZN: Conducted experiment, data collection, and revised the manuscript. DQ: Data extraction and Statistical analyses. DQ: Drafted the manuscript. All authors have read and approved the final manuscript.
